# Surgical Procedure among Patients with Foreign Body Obstruction in Food Passage in a Tertiary Care Hospital: A Descriptive Cross-sectional Study

**DOI:** 10.31729/jnma.5704

**Published:** 2021-05-31

**Authors:** Krishna Chandra Rijal, Krishna Prasad Koirala, Ashish Khadgi

**Affiliations:** 1 Department of Otorhinolaryngology-Head and Neck Surgery, Manipal College of Medical Sciences, Pokhara, Nepal

**Keywords:** *esophagoscopy*, *esophagus*, *foreign bodies*

## Abstract

**Introduction:**

Foreign body is any object in a region it is not meant to be, where it can cause harm if immediate medical attention is not sought. Its removal by surgical procedure is one of the commonest surgeries done. The objective is to find out prevalence of the patients who underwent operation for foreign bodies obstruction in food passage in the Department of Otorhinolaryngology-Head and Neck Surgery in a tertiary care centre.

**Methods:**

A descriptive cross-sectional study conducted among patients operated in the Department of Otorhinolaryngology and Head and Neck Surgery of a tertiary care center from August 2014 to May 2017. Ethical approval was received from the Institutional Review Committee of the Institute. Convenience sampling method was used. Statistical Package for the Social Sciences was used for analysis. Point estimate at 95% Confidence Interval was calculated along with frequency and proportion for binary data.

**Results:**

Out of total 700 patients having operation in department, 115 (16.42%) (95% Confidence Interval = 13.67-19.16) had operation for foreign body obstruction in the food passage. Among 115 patients, 62 (53.90%) were males and 53 (46.10%) were females. Most common foreign bodies ingested in children was coin 17 (14.78%) and bone chips 40 (34.78%) in adults. Cricopharynx 90 (78.26%) is the commonest site for foreign body lodgement.

**Conclusions:**

Prevalence of the patients who underwent operation for foreign bodies obstruction in food passage in a tertiary care hospital is high. Their removal by rigid oesophagoscopy is one of the commonest surgical procedures done in tertiary care center.

## INTRODUCTION

Foreign body is an object that has entered the body by accident or design. Foreign bodies are not uncommon in developing countries like Nepal. Ingested foreign bodies are one of the common emergencies faced by otorhinolaryngologists and needs early interventions otherwise leads to increase morbidity and mortality. They form the leading cause of death in children under the age of one year and fourth leading cause of death in the age group 1-6 years.^1^

Foreign body is common in children because they are naturally curious about their surroundings and about body orifices hence inclined to place foreign bodies in ear, nose or oral cavity.^2^

Rigid esophagoscopy remains the best mode of treatment for the removal of ingested foreign bodies.^3,4^

The objective is to find out prevalence of the patients who underwent operation for foreign bodies obstruction in food passage in the Department of Otorhinolaryngology and Head and Neck Surgery in a tertiary care centre.

## METHODS

This is a descriptive cross-sectional study conducted among patients operated in the Department of Otorhinolaryngology, Head and Neck Surgery, Manipal College of Medical Sciences from August 2014 to May 2017 from the medical records of patients. Ethical approval was received from the Institutional Review Committee of the Institute. The complete data of the patients having operation in the department during study period irrespective of age and sex were included in the study. The patients having incomplete or missing data were excluded from the study. The convenience sampling method was used.

The sample size was calculated by using formula,


$$\begin{array}{ll} \text{n} & \text{= }{\text{Z}}^{\text{2}}\times p\times q\;/{\text{e}}^{\text{2}}\\ & \text{= }{\left(1.96\right)}^{\text{2}}\times (0.5)\times (0.5)/{(\text{0.04)}}^{\text{2}}\\ & \text{= }600 \end{array}$$

Where,

n = minimum required sample size
Z = 1.96 at 95% Confidence Interval (CI)
p = prevalence taken as 50% for maximum sample size
q = 1-p
e = margin of error, 4%

The calculated sample size was 600. Taking nonresponse rate 10%, the sample size became 660. However, the total sample size of 700 was taken.

The data of the patients were retrieved from operative register of Otorhinolaryngology, Head and Neck Surgery. Statistical analysis of the study was done for various parameters like incidence, age, sex, types of foreign bodies, anatomical site of lodgment of foreign bodies and surgical intervention taken for their removal with appropriate statistical method.

Statistical Package for the Social Sciences (SPSS) program was used for analysis.

## RESULTS

**Figure 1.**
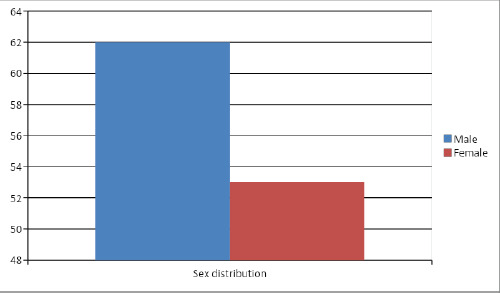
Sex distribution of the patients with foreign body food passage.

Out of total 700 patients having operation, 115 (16.42%) (95% Confidence Interval = 13.67-19.16) had operation for foreign body obstruction in the food passage. A total of 115 patients included in the study, 62 (53.90%) were males and 53 (46.10%) were females (Figure 1).

The minimum age of presentation was 10 months and maximum age was 92 years with a mean age 46.41 of years. Coin 17 (14.78%) was the most common foreign bodies ingested in children and bone chips 40 (34.78%) was the most common foreign bodies ingested in adult (Table 1).

**Table 1:** Types of foreign bodies in different age group (n = 115).

Types of FBs	Age (yrs.) n (%)								Total n (%)
	0-10	11-20	21-30	31-40	41-50	51-60	61-70	>71	
**Bone**	1 (0.86)	1 (0.86)	7 (6.08)	9 (7.82)	5 (4.34)	9 (7.82)	5 (4.34)	3 (2.60)	40 (34.78)
**Meat bolus**				1 (0.86)	2 (1.73)	2 (1.73)	14 (12.17)	12 (10.43)	31 (26.95)
**Cartilage**			1 (0.86)	1 (0.86)					2 (1.73)
**Meat + bone**					2 (1.73)	4 (3.47)	4 (3.47)	2 (1.73)	12 (10.43)
**Meat + cartilage**				1 (0.86)		2 (1.73)	2 (1.73)		5 (4.34)
**Coin**	16 (13.91)	1 (0.86)							17 (14.78)
**Artificial denture**				1 (0.86)		1 (0.86)		1 (0.86)	3 (2.60)
**Fish bone**					1(0.86)				1 (0.86)
**Metallic star**	1 (0.86)								1 (0.86)
**Metallic sharpener**	1 (0.86)								1 (0.86)
**Metallic pin**				1 (0.86)					1 (0.86)
**Battery**		1 (0.86)							1 (0.86)
**Total**	19	3	8	14	10	18	25	18	1115
** **	(16.52)	(2.60)	(6.95)	(12.17)	(8.69)	(15.65)	(21.73)	(15.65)	(100)

Cricopharynx 90 (78.26%) was the most common site of foreign body lodgment follwed by oesophagus 13 (11.30%) and pyriform sinus 11 (9.56%) (Table 2).

**Table 2:** Site of foreign body lodgement (n = 115).

Sites→	Vallecula n (%)	Pyriform Sinus n (%)		Cricopharynx n (%)	Oesophagus n (%)			Total n (%)
Types of FBs↓		Right	Left		Upper	Mid	Lower	
**Bone**		6 (5.21)	3 (2.60)	31 (26.95)				40 (34.78)
**Meat bolus**				18 (15.65)	3 (2.60)	4 (3.47)	6 (5.21)	31 (26.95)
**Cartilage**				2 (1.73)				2 (1.73)
**Meat + bone**				12 (10.43)				12 (10.43)
**Meat + cartilage**				5 (4.34)				5 (4.34)
**Coin**				17 (14.78)				17 (14.78)
**Artificial denture**		1 (0.86)		2 (1.73)				3 (2.60)
**Fish bone**	1 (0.86)							1 (0.86)
**Metallic star**				1 (0.86)				1 (0.86)
**Metallic sharpener**				1 (0.86)				1 (0.86)
**Metallic pin**		1 (0.86)						1 (0.86)
**Battery**				1 (0.86)				1 (0.86)
**Total**	1 (0.86)	11 (9.56)		90 (78.26)	13 (11.30)			115 (100)

**Figure 2.**
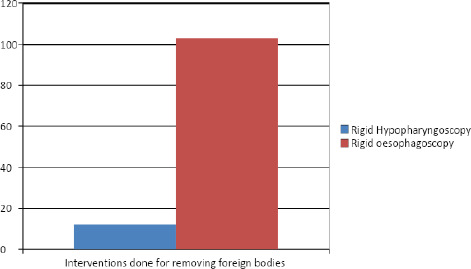
Interventions done for removing foreign bodies.

Rigid Oesophagoscopy was carried out in 103 (90%) patients and rigid hypopharyngoscopy was carried out in 12 (10%) patients (Figure 2).

## DISCUSSION

Foreign body is a common ENT emergency accounting for about 11% of the cases in ENT emergency.^5^ As reported by Bhatta R, et al.^6^ digestive tract is the commonest site for foreign body impaction after ear, nose and respiratory tract.

There were 115 cases, the patients underwent operation for foreign body obstruction in the food passage which is more common than other ENT foreign bodies. Gupta P, et al.^1^ found, 90% and 10% foreign body in food passage and air passage respectively while Showkat SA, et al.^7^ also have similar findings, showing ingested foreign body is more common which correlate with our study. Our study showed male predominance with Male: Female ratio of 1.55:1 which is also supported by other studies.^2,8,9^

The youngest patient was 10-month-old with coin at the level of cricopharynx whereas the oldest patient was 92-year-old male with meat bolus in the cricopharynx. The most common age group in our study was 61-70 years with 44.98% of patients but most studies showed it is common in paediatric age group. 10 months-10 years of age group ranked second most common age group as a sequel to natural proclivity to put things in their mouth in children. Ingested foreign bodies were located at cricopharynx in 90 (78.92%) of 115 patients. This is owing to poor peristalsis, sphincter action, and narrow diameter of cricopharynx. About 80% of the ingested foreign body are held up in cricopharynx as reported in different literature^7,10^ showing cricopharynx is the commonest site of digestive tract foreign body, which is similar to the findings of our study. We observed bone chips 40 (39.63%) to be the commonest types of FB in adult and elderly age group and coins 17 (38.49%) in children which is also supported by other literatures.^8,11^

Rigid oesophagoscopy was carried out in 103 patients and rigid hypopharyngoscopy was carried out in 12 patients. Rigid esophagoscopy for the removal of foreign bodies digestive tract remains the best mode of treatment.^3,4^

Since this is a descriptive cross-sectional and conducted in a single tertiary care center, the findings of the study cannot be generalized to whole population. Foreign bodies obstruction in food passage are common presentation in the Department of Otorhinolaryngology, Head and Neck Surgery. More study should be conducted for the proper management of the patients having foreign body obstruction in food passage.

## CONCLUSIONS

Prevalence of the patients who underwent operation for foreign bodies obstruction in food passage in a tertiary care hospital is high. Foreign body obstruction is common in all age group. Cricopharynx is the commonest site of impaction and bone piece is the commonest type of foreign body. Their removal by rigid esophagoscopy is one of the commonest surgical procedures done in a tertiary care center.
